# Public Opinion on Firearm Injury Prevention Proposals in California

**DOI:** 10.1001/jamanetworkopen.2019.18786

**Published:** 2020-01-08

**Authors:** Rocco Pallin, Amanda Charbonneau, Nicole Kravitz-Wirtz, Garen J. Wintemute

**Affiliations:** 1 Violence Prevention Research Program, Department of Emergency Medicine, University of California, Davis, School of Medicine, Sacramento, California

## Abstract

This survey study assesses public opinion on 2 firearm injury prevention proposals in California overall and by firearm ownership status.

## Introduction

Agreement between firearm owners and nonowners on many firearm violence prevention proposals is more common than typically recognized.^1^

This survey study assessed public opinion on 2 proposals in California, overall and by firearm ownership status. One proposal is an amnesty for high-capacity ammunition magazines. In 2016, voters approved (63.1% in favor) a ban on possession that has since been challenged in federal court.^2^ Research suggests that restricting high-capacity magazines and weapons that use them may reduce firearm violence.^3^

The second proposal would prohibit firearm purchase and possession by persons with multiple recent convictions for driving under the influence of alcohol or drugs (DUI). There is substantial evidence showing that alcohol misuse is associated with increased risk for violence, including among firearm owners.^4,5^

## Methods

The California Safety and Well-being Survey was designed by us and administered online in 2018 by Ipsos Public Affairs, LLC; detailed methods are presented elsewhere.^6^ Respondents were California residents aged 18 years and older from the Ipsos KnowledgePanel, a probability-based internet panel sourced using address-based sampling. Respondents provided online informed consent by initiating the survey, and their answers were weighted to represent California’s adult population. We calculated weighted proportions with 95% confidence intervals for each measure or crosstabulation using Stata SE software version 15.1 (StataCorp).

This study followed the American Association for Public Opinion Research (AAPOR) reporting guideline and was approved by the institutional review board at the University of California, Davis.

## Results

Of 5232 eligible panel members with baseline profile information, 2558 completed the survey (48.9% completion rate). Respondents were informed of the state’s existing high-capacity magazine ban, after which a majority (62.3%; 95% CI, 59.2%-65.4%) indicated support for “an amnesty program that allows people to turn in ammunition magazines that hold more than 10 bullets, no questions asked” (Table 1). No difference between firearm owners and nonowners was observed; support among firearm owners decreased as the number of guns owned increased: among owners of 1 to 3 guns, 61.1% (95% CI, 52.4%-69.2%) supported amnesty compared with 26.7% (95% CI, 11.2%-51.3%) of owners of 10 or more guns. More owners without high-capacity magazines (53.2%; 95% CI, 45.4%-60.8%) supported amnesty than those with high-capacity magazines (41.4%; 95% CI, 23.5%-62.0%).

**Table 1.** Respondent Support for an Amnesty for High-Capacity Magazines^a^

Characteristic	Support		Oppose		Do Not Know		Total No.
	Unweighted No.^b^	Weighted % (95% CI)	Unweighted No.	Weighted % (95% CI)	Unweighted No.	Weighted % (95% CI)	
Total	1657	62.3 (59.2-65.4)	407	17.6 (15.3-20.2)	407	20.1 (17.5-22.9)	2471
Firearm ownership status							
Owner	234	51.2 (44.0-58.3)	112	29.1 (22.9-36.2)	80	19.7 (14.5-26.2)	426
Lives with owner	160	58.0 (47.9-67.5)	43	16.3 (10.3-24.7)	38	25.7 (17.3-36.3)	241
No guns in home	1228	66.4 (62.5-70.1)	235	15.3 (12.7-18.4)	258	18.3 (15.3-21.7)	1721
Age, y							
18-29	73	45.7 (36.2-55.5)	28	17.4 (11.2-25.8)	48	36.9 (27.9-46.9)	149
30-44	281	65.3 (58.9-71.2)	84	19.4 (14.7-25.1)	80	15.3 (11.2-20.5)	445
45-59	419	63.9 (58.2-69.2)	111	17.4 (13.4-22.1)	121	18.8 (14.7-23.7)	651
≥60	884	68.3 (64.0-72.2)	184	16.3 (13.3-19.9)	158	15.4 (12.4-19.1)	1226
Sex							
Male	691	62.0 (57.2-66.7)	204	19.5 (16.0-23.5)	164	18.5 (14.8-22.8)	1059
Female	966	62.6 (58.4-66.6)	203	15.9 (13.0-19.2)	243	21.5 (18.2-25.4)	1412
Race/ethnicity							
White, non-Hispanic	1024	64.8 (60.9-68.5)	209	15.3 (12.8-18.1)	207	19.9 (16.7-23.7)	1440
Hispanic	375	54.6 (47.9-61.1)	152	24.5 (19.2-30.6)	138	21.0 (15.9-27.1)	665
Black, non-Hispanic	96	69.4 (54.8-80.8)	6	4.2 (1.6-10.9)	19	26.4 (15.4-41.5)	121
Other, non-Hispanic	162	67.3 (59.6-74.1)	40	15.8 (10.9-22.4)	43	16.9 (12.0-23.3)	245
Education							
No college	201	49.6 (43.0-56.2)	87	21.8 (16.9-27.8)	94	28.6 (22.7-35.2)	382
Some college	539	62.4 (57.4-67.2)	167	19.4 (15.7-23.8)	160	18.2 (14.6-22.3)	866
Bachelor's degree or higher	917	74.4 (70.4-78.1)	153	11.8 (9.4-14.8)	153	13.8 (10.9-17.2)	1223
Political ideology							
Liberal	714	77.0 (71.9-81.5)	82	10.6 (7.7-14.5)	78	12.3 (8.9-16.8)	874
Moderate	475	56.9 (51.0-62.6)	123	18.2 (14.1-23.2)	140	24.9 (20.0-30.6)	738
Conservative	384	50.4 (44.8-55.9)	188	28.2 (23.3-33.7)	151	21.4 (17.1-26.4)	723
Member or generally supportive of National Rifle Association or other firearm rights organization							
No	1378	67.9 (64.3-71.3)	204	12.8 (10.5-15.5)	277	19.3 (16.5-22.6)	1859
Yes	233	40.5 (34.2-47.1)	196	38.1 (31.8-44.9)	112	21.4 (16.2-27.7)	541
Among gun owners							
No. of guns owned							
1-3	174	61.1 (52.4-69.2)	56	20.1 (14.6-26.9)	43	18.8 (12.5-27.2)	273
4-9	50	35.6 (23.9-49.4)	30	42.7 (27.4-59.6)	28	21.7 (11.9-36.2)	108
≥10	9	26.7 (11.2-51.3)	23	56.6 (35.1-75.8)	6	16.3 (6.6-34.9)	39
Owns high-capacity magazine (>10 rounds)							
No	215	53.2 (45.4-60.8)	91	27.1 (20.8-34.5)	67	19.5 (13.8-26.6)	373
Yes	19	41.4 (23.5-62.0)	19	41.6 (23.0-63.0)	11	16.9 (8.0-32.2)	49

^a^ Respondents were shown the statement, “It is illegal in California to buy or sell ammunition magazines that hold more than 10 bullets, or to bring them into the state. It may soon be illegal to have them.” They were then asked, “Would you support or would you oppose an amnesty program that allows people to turn in ammunition magazines that hold more than 10 bullets, no questions asked?”

^b^ Sixty-nine Spanish-language respondents (30.4%) are missing from these data owing to a survey translation error.

After reading a definition of DUI and, for a randomized subset of respondents, a statement about the association between DUI and risk of future violence, majorities of all respondents (67.9%; 95% CI, 64.9%-70.8%), those living with firearm owners (66.5%; 95% CI, 56.2%-75.5%), and nonowners (72.3%; 95% CI, 68.8%-75.6%) supported “a law that prevents someone from buying a gun for 5 years if they have had 2 or more DUI convictions in 5 years” (Table 2). Half of firearm owners (50.0%; 95% CI, 42.8%-57.1%) indicated support for such a law. Support did not vary significantly by self-reported alcohol use or by exposure to the statement about DUI and violence.

**Table 2.** Respondent Support for DUI-Based Prohibition on Firearm Purchase and Possession^a^

Characteristic	Support		Oppose		Do Not Know		Total No.
	Unweighted No.	Weighted % (95% CI)	Unweighted No.	Weighted % (95% CI)	Unweighted No.	Weighted % (95% CI)	
Total	1803	67.9 (64.9-70.8)	363	16.2 (14.0-18.8)	380	15.9 (13.7-18.3)	2546
Firearm ownership status							
Owner	248	50.0 (42.8-57.1)	111	31.4 (25.0-38.6)	69	18.6 (13.2-25.7)	428
Lives with owner	177	66.5 (56.2-75.5)	21	11.2 (5.9-20.1)	44	22.3 (14.7-32.2)	242
No guns in home	1337	72.3 (68.8-75.6)	214	13.9 (11.4-16.9)	240	13.8 (11.4-16.6)	1791
Age, y							
18-29	108	65.5 (55.8-74.1)	15	9.4 (5.3-16.1)	33	25.0 (17.4-34.6)	156
30-44	319	65.5 (59.2-71.3)	81	21.7 (16.6-27.9)	75	12.8 (9.6-16.9)	475
45-59	472	68.9 (63.5-73.9)	109	17.7 (13.7-22.6)	96	13.4 (10.2-17.4)	677
≥60	904	70.8 (66.6-74.7)	158	13.7 (10.9-17.0)	176	15.5 (12.4-19.2)	1238
Sex							
Male	715	64.1 (59.3-68.6)	205	20.7 (17.1-24.9)	164	15.2 (12.0-19.1)	1084
Female	1088	71.4 (67.6-75.0)	158	12.2 (9.6-15.2)	216	16.4 (13.6-19.7)	1462
Race/ethnicity							
White, non-Hispanic	1017	65.8 (62.0-69.5)	192	16.1 (13.4-19.4)	232	18.0 (15.1-21.4)	1441
Hispanic	518	68.1 (61.8-73.7)	126	19.3 (14.7-24.9)	94	12.7 (9.0-17.5)	738
Black, non-Hispanic	85	59.8 (45.9-72.2)	16	15.3 (7.9-27.6)	20	24.9 (14.2-39.9)	121
Other, non-Hispanic	183	74.9 (67.5-81.0)	29	11.3 (7.3-17.1)	34	13.8 (9.2-20.2)	246
Education							
No college	256	58.3 (51.9-64.4)	91	21.9 (17.0-27.7)	77	19.8 (15.2-25.5)	424
Some college	628	72.1 (67.5-76.3)	119	13.9 (11.0-17.5)	135	14.0 (10.9-17.8)	882
Bachelor's degree or higher	919	73.7 (69.6-77.4)	153	12.7 (9.9-16.2)	168	13.6 (10.9-16.7)	1240
Political ideology							
Liberal	699	75.2 (70.1-79.7)	72	11.2 (7.9-15.6)	113	13.6 (10.4-17.6)	884
Moderate	546	67.0 (61.4-72.1)	106	15.5 (11.8-20.1)	118	17.5 (13.5-22.4)	770
Conservative	471	61.6 (56.0-66.9)	158	23.2 (18.7-28.4)	116	15.2 (11.6-19.7)	745
Member or generally supportive of National Rifle Association or other firearm rights organization							
No	1453	72.4 (69.0-75.5)	197	12.6 (10.3-15.2)	274	15.1 (12.6-17.9)	1924
Yes	304	54.5 (47.8-61.1)	151	28.3 (22.6-34.8)	88	17.2 (12.6-22.9)	543
Risk for alcohol use disorder^b^							
No risk	939	66.9 (62.5-71.0)	175	15.6 (12.6-19.2)	190	17.5 (14.3-21.3)	1304
Low risk	707	70.4 (65.8-74.7)	151	17.0 (13.5-21.1)	148	12.6 (9.9-16.0)	1006
High risk	108	68.5 (57.1-78.0)	23	14.7 (7.9-25.7)	29	16.8 (10.8-25.2)	160

Abbreviation: DUI, driving under the influence of alcohol or drugs.

^a^ Respondents were either shown the statement “DUI means driving under the influence of alcohol or drugs. Gun owners who have been convicted of DUI are 4 times as likely as other gun owners to commit violent crimes in the future” or the statement “DUI means driving under the influence of alcohol or drugs.” They were then asked, “Would you support or would you oppose a law that prevents someone from buying a gun for 5 years if they have had 2 or more DUI convictions in 5 years?” There was no statistically significant difference in support among those who received the more detailed statement before responding to the question compared with the shorter statement. These results are for the total survey population who responded to this question regardless of preamble.

^b^ Respondents' alcohol consumption was measured by number of drinks in the past week and categorized based on the National Institutes of Health definition of risk for alcohol use disorder.

Variation in support by demographic characteristics and political ideology was similar for both proposals. Half of respondents supported both proposals (50.5%; 95% CI, 47.3%-53.7%), and 6.5% (95% CI, 5.1%-8.3%) opposed both.

## Discussion

Most respondents supported an amnesty for high-capacity ammunition magazines and a prohibition based on DUI convictions, including at least half of firearm owners. Support for one proposal but not the other was common, but opposition to both was rare, suggesting that we did not simply capture respondents’ general opinions on firearm regulation. Nationally, support for firearm policies is also high, and gaps between owners and nonowners are frequently small.^1^

Our findings have limitations. First, some reported opposition to magazine amnesty may represent general opposition to the magazine ban. Second, survey research is subject to nonresponse error and social desirability bias. Third, given California’s relatively low rates of and strict regulations on firearm ownership, our results may not be generalizable. Replication in other states is recommended.
